# Soft‐Template Electropolymerization from Triphenylamine‐Based Monomers: From Vertically Aligned Nanotubes to Nanomembranes

**DOI:** 10.1002/open.202500050

**Published:** 2025-04-01

**Authors:** Khady Diouf, Alioune Diouf, Abdoulaye Dramé, Frédéric Guittard, Thierry Darmanin

**Affiliations:** ^1^ Université Cheikh Anta Diop Faculté des Sciences et Techniques Département de Chimie B.P. 5005 Dakar Sénégal; ^2^ Université Côte d'Azur NICE Lab 06100 Nice France

**Keywords:** Conjugated polymers, Electropolymerization, Nanomembrane, Nanotubes, Soft-template

## Abstract

We report a bioinspired approach to tune surface nanostructures by soft‐template electropolymerization in micellar condition. Monomers highly favoring π‐stacking interactions are particularly interesting for depositing in one direction resulting in vertically aligned nanotubes. Here, for inducing very strong *π*‐stacking interactions, a triphenylamine building block was selected and substituted by two substituents of different electronegativity (fluorine F and methoxy OMe). These synthons were di‐substituted with various fully conjugated thiophene and carbazole derivatives. Here, all the monomers have high electrodeposition capacity except the monomers with thiophene in 3‐position. Confirming previous works, electrochemical analyses in the electrodeposited films show the presence of monomers but with significant difference as a function of the used monomer. The surface structures are highly depending on the monomer structure while the depositions at constant potential lead to more ordered structures. With some of these monomers, densely packed nanotubes are created and their merger at high deposition charge, leading to nanomembranes. Their hydrophobicity and oleophobicity are also investigated and extremely various. Such materials could be used in the future in practical applications such as in oil/water separation membranes or in water‐harvesting systems.

## Graphical abstract

Tunable surface structures are prepared by soft‐template electropolymerization in micellar solution. Various monomers were synthesized from a triphenylamine core. Fully conjugated monomers are studied for favoring extremely strong π‐stacking interactions. Most of these monomers have high electrodeposition capacity. The presence of monomers in electrodeposited films was confirmed by electrochemical analyses. The monomer has a huge influence on the surface structures. Densely packed nanotubes were observed with their merger at high deposition charge, leading to nanomembranes. Their hydrophobicity and oleophobicity are also investigated and are extremely various.

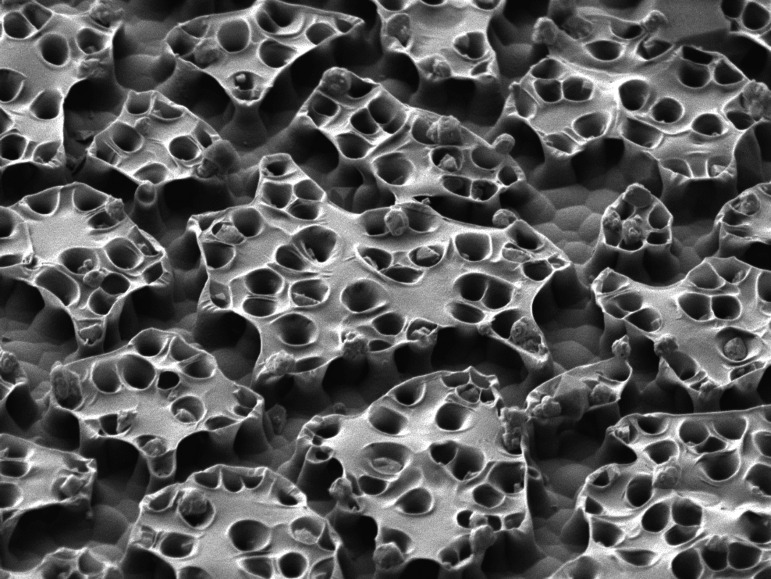

## Introduction

Controlling the shape of surface nanostructures is fundamental for numerous concrete applications such as in optics,[^1^, ^2^] magnetics,^[3]^ biomedicine,[^4^, ^5^] energy systems^[6]^ or in batteries.^[7]^ This is particularly true for the wetting properties.[^8^, ^9^, ^10^] Bioinspired strategies revealed the key role of surface structures in superhydrophobic or superoleophobic surfaces.[^11^, ^12^, ^13^, ^14^, ^15^]

Electropolymerization is a very fast electrochemical process to deposit conducting polymer films of controllable thickness by oxidizing a monomer in solution.[^16^, ^17^, ^18^, ^19^] By tuning the monomer structure, various structures such as nanopheres, nanosheets or nanofibers can be formed but especially non‐porous structures.[^20^, ^21^] For their wetting properties, nanotubes were found to be an excellent choice for the control of both the surface hydrophobicity and the water adhesive forces.^[22]^ Porous structures like nanotubes can be prepared by electropolymerization using hard templates (anodized aluminum oxide (AAO) membranes, as example). However, numerous templates are necessary to create nanotubes of various dimension (diameter, height or pitch), and these templates have to be removed after use.[^23^, ^24^, ^25^, ^26^]

To replace hard templates, soft templates, consisting in gas bubbles or micelles in solution, can be employed. If the electropolymerization process is performed directly in water (H_2_O), gas bubbles can be formed on the substrate during the process.[^27^, ^28^, ^29^, ^30^, ^31^, ^32^] Oxygen bubbles (O_2_) can be formed from H_2_O (water oxidation: 2H_2_O→O_2_ + 4H^+^+ 4e^–^) if a potentiostatic process is used such as at constant potential or current. Hydrogen bubbles (H_2_) can be also formed from H_2_O (water reduction: 2H_2_O + 2e^–^→H_2_ + 2OH^–^) if a potentiodynamic process is used such as by cyclic voltammetry and pulsed electropolymerization, but only if the potential range is sufficiently large.^[33]^

Most of the monomers being not soluble in H_2_O, a solvent of low‐water solubility such as dichloromethane (CH_2_Cl_2_) or chloroform (CHCl_3_) can be used instead as soon as H_2_O is present in solution.[^34^, ^35^, ^36^, ^37^] The process is different because micelles can be formed in solution prior electropolymerization.^[38]^ The micelles can be stabilized by the electrolyte especially if this one acts as a surfactant such as tetrabutylammonium perchlorate (Bu_4_NClO_4_).

To form vertically aligned nanotubes by soft‐template electropolymerization, it is fundamental to favor the polymer growth on the substrate in one‐direction (1–D).^[39]^ Highly conjugated monomers such as 3,4‐(1,2‐phenylenedioxy)thiophene (PheDOT) or 3,4‐(1,2‐naphthylenedioxy)thiophene (NaphDOT) gave exceptional results because the polymer deposition is favored in the direction perpendicular to the molecule by *π*‐stacking interactions.

Triphenylamine was found to be an excellent core leading to intense *π*‐stacking interactions while significantly involved in the initial electron transfer process (oxidation) to form radical cations species during the electropolymerization process.[^40^, ^41^, ^42^] Triphenylamine tri‐substituted with different thiophene and carbazole derivatives were studied and extremely long tubes were obtained particularly with carbazole in *para*‐position.^[41]^ Indeed, it was calculated that the molecules are not fully flat because steric hindrances result in some distortion between the conjugated units. As expected, the less distortion was found with carbazole in *para*‐position and the worst with carbazole in *ortho*‐position.

**Scheme 1:**
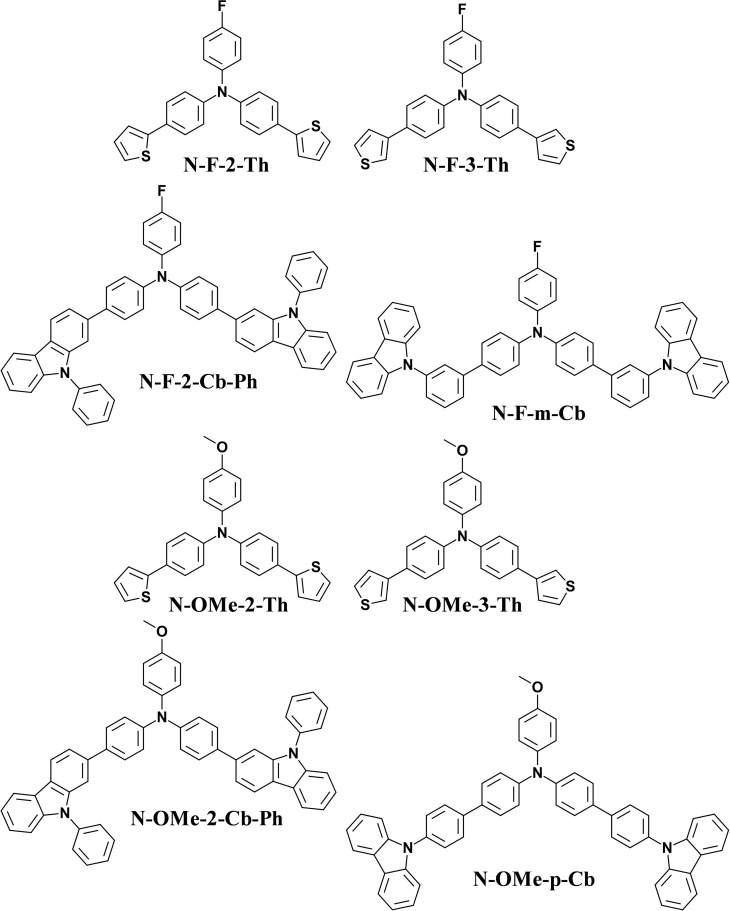
Original triphenylamine‐based monomers investigated in this manuscript.

Here, original di‐substituted triphenylamine‐based monomers were synthesized and investigated (Scheme 1). On the one hand, two substituents of different mesomeric effect and electronegativity were tested: fluoride (F) and methoxy (OMe). Indeed, because the substituents are directly bound on the monomer, it is expected a much higher decrease of reactivity with F compared to OMe especially because F has the highest electronegativity. For example, thiophene or indole with various substituents were already investigated in the literature.^[43]^ On the other hand, for the di‐substitution various conjugated thiophene and carbazole‐based derivatives in order to compare with previous works.^[41]^ Thiophene is a highly polymerizable molecule compared to carbazole. Electropolymerization of thiophene derivatives is known to occur preferentially at position 2 and carbazole ones at positions 3, and the polymer chains are much longer with thiophene derivatives while only extremely short oligomers (dimers, trimers…) are expected with carbazole. But the fact that these molecules are fully conjugated, the electropolymerization should occur one time on all these molecules, even if there are not completely planar.^[41]^ The polymer deposition would be highly increased by *π*‐stacking interactions while limiting polymerization. These monomers investigated are fully conjugated for favoring the formation of nanotubes but it could be envisaged in the future to add functional groups such as hydroxyl, carboxylic acids or azide functions.^[44]^ However, it will be necessary to verify the influence on the formation of nanotubes.^[45]^

Here, the monomers have excellent electrodeposition capacity, excepted the monomers with thiophene in 3‐position. In the electrodeposited polymer films, the presence of monomer is detected confirming previous works but here the proportion of oligomerization is dependent on the used monomer. For the surface structures, the more interesting results are obtained at constant potential. With some of these monomers, densely packed nanotubes are formed and it is observed the merge of the tubes at high deposition charge leading to nanomembranes, which is extremely rare in literature.

## Materials and Method

### Monomer Synthesis and Characterization

**Scheme 2:**
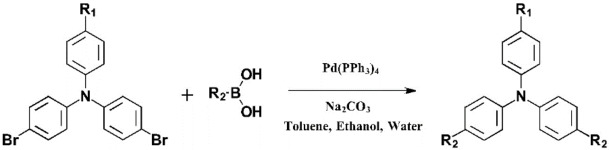
Synthesis way to monomers.

All chemicals were purchased from Merck while all reactants were purchased form TCI. These original monomers were obtained using two Suzuki reactions (Scheme 2).^[46]^ Two dibromides (*N*,*N*‐bis(4‐bromophenyl)‐4‐fluoroaniline and 4,4′‐dibromo‐4′′‐methoxytriphenylamine) were tested as starting reactants. Six boronic acids including two thiophene derivatives (2‐thiopheneboronic acid, 3‐thiopheneboronic acid) and four carbazole derivatives (4‐(9*H*‐carbazol‐9‐yl)phenylboronic acid, 3‐(9*H*‐carbazol‐9‐yl)phenylboronic acid, 2‐(9*H*‐carbazol‐9‐yl)phenylboronic acid and 9‐phenylcarbazole‐2‐boronic acid). However, the monomers obtained with sufficient yield are the monomers represented in Scheme 1.

More precisely, dibromide (2.0 mmol, 1.0 eq.), boronic acid (5.0 mmol, 2.5 eq.), tetrakis(triphenylphosphine)‐palladium(0) (0.1 mmol, 0.1 eq.) and sodium carbonate (4.0 g) were added to a mixture of toluene (100 mL), ethanol (20 mL) and water (10 mL). After refluxing for 3 days, the solvents were evaporated and the crude products were purified by column chromatography (silica gel; eluent: petroleum ether/chloroform).

NMR spectra were recorded with a 400 MHz ultra‐blinded spectrometer equipped with an AVANCE III Nano HD electronic console. The proton and carbon frequencies were 400.13 MHz and 100.63 MHz, respectively.

4‐fluoro‐*N*,*N*‐bis(4‐(thiophen‐2‐yl)phenyl)aniline (**N‐F‐2‐Th**).

Yield: 17 %; ^1^H NMR (400 MHz, CDCl_3_) δ 7.50 (d, *J*=8.7 Hz, 2H), 7.24 (d, *J*=4.4 Hz, 2H), 7.13 (dd, *J*=9.0, 4.8 Hz, 1H), 7.10 – 7.04 (m, 3H), 7.00 (t, *J*=8.6 Hz, 1H); ^13^C NMR (400 MHz, CDCl_3_) δ 159.45, 157.02, 145.82, 143.08, 142.20, 132.50, 127.85, 126.98, 125.83, 125.77, 123.13, 122.55, 121.34, 115.37, 115.15.

4‐fluoro‐*N*,*N*‐bis(4‐(thiophen‐3‐yl)phenyl)aniline (**N‐F‐3‐Th**).

Yield: 99 %; ^1^H NMR (400 MHz, CDCl_3_) δ_H_ : 7.49 (d, J=8.6 Hz, 4H), 7.37 (dd, J=4.3, 2.9 Hz, 6H), 7.12(dd, J=17.7, 8.8 Hz, 6H), 7.00 (t, J=8.6 Hz, 2H).

^13^ C NMR (101 MHz, CDCl_3_) δ_C_ : 160.31, 157.89, 146.69, 143.49, 141.80, 130.27, 127.27, 126.62, 126.13, 123.64, 119.31, 116.30, 116.08.

4‐fluoro‐*N*,*N*‐bis(4‐(9‐phenyl‐9*H*‐carbazol‐2‐yl)phenyl)aniline (**N‐F‐2‐Cb‐Ph**).

Yield: 24 %; ^1^H NMR (400 MHz, CDCl_3_) δ 8.17 (t, *J*=7.6 Hz, 2H), 7.63 (d, *J*=15.8 Hz, 4H), 7.59 – 7.46 (m, 3H), 7.41 (d, *J*=4.2 Hz, 2H), 7.31 (d, *J*=6.2 Hz, 1H), 7.20 – 7.07 (m, 2H), 6.98 (t, *J*=6.9 Hz, 1H); ^13^C NMR (400 MHz, CDCl_3_) δ 147.71, 147.02, 146.83, 141.55, 141.44, 140.29, 138.84, 137.65, 136.07, 135.87, 129.94, 129.91, 129.28, 128.24, 128.19, 127.55, 127.51, 127.27, 127.22, 126.74, 126.65, 126.62, 126.54, 125.89, 125.86, 123.70, 123.61, 123.42, 123.17, 122.70, 122.46, 122.30, 122.26, 120.53, 120.44, 120.30, 120.23, 120.09, 120.04, 119.22, 116.31, 116.22, 116.07, 116.00, 109.77, 109.75, 108.67, 107.66.

*N*‐(3′‐(9*H*‐carbazol‐9‐yl)‐[1,1′‐biphenyl]‐4‐yl)‐3′‐(9*H*‐carbazol‐9‐yl)‐N‐(4‐fluorophenyl)‐[1,1′‐biphenyl]‐4‐amine (**N‐F‐m‐Cb**).

Yield: 51 %; ^1^H NMR (400 MHz, CDCl_3_) δ 8.17 (d, *J*=7.7 Hz, 2H), 7.78 (s, 1H), 7.70 – 7.62 (m, 2H), 7.53 (dd, *J*=10.2, 7.7 Hz, 3H), 7.44 (dt, *J*=14.1, 7.6 Hz, 4H), 7.30 (t, *J*=7.4 Hz, 2H), 7.17 (d, *J*=8.6 Hz, 3H); ^13^C NMR (400 MHz, CDCl_3_) δ 147.37, 143.21, 142.43, 140.87, 138.23, 134.21, 130.24, 127.95, 127.16, 127.08, 125.94, 125.58, 125.40, 125.20, 123.58, 123.39, 120.33, 119.94, 116.47, 116.24, 109.80.

4‐methoxy‐*N*,*N*‐bis(4‐(thiophen‐2‐yl)phenyl)aniline (**N‐OMe‐2‐Th**).

Yield: 11 %; ^1^H NMR (400 MHz, CDCl_3_) δ 9.44 (d, *J*=8.6 Hz, 4H), 9.18 (d, *J*=4.3 Hz, 4H), 9.12 – 8.95 (m, 8H), 8.84 (d, *J*=9.0 Hz, 2H), 5.78 (s, 3H); ^13^C NMR (400 MHz, CDCl_3_) δ 155.53, 146.13, 143.30, 139.04, 127.20, 126.94, 126.47, 125.70, 122.90, 121.93, 121.13, 113.89, 54.48.

4‐methoxy‐*N*,*N*‐bis(4‐(thiophen‐3‐yl)phenyl)aniline (**N‐OMe‐3‐Th**).

Yield: 35 %; ^1^H NMR (400 MHz, CDCl_3_) δ 7.47 (d, *J*=8.6 Hz, 4H), 7.36 (s, 6H), 7.11 (dd, *J*=14.5, 8.7 Hz, 6H), 6.88 (d, *J*=8.9 Hz, 2H), 3.82 (s, 3H); ^13^C NMR (400 MHz, CDCl_3_) δ 155.35, 145.97, 140.93, 139.34, 128.59, 126.34, 126.10, 125.10, 121.99, 118.04, 113.83, 54.47.

4‐methoxy‐*N*,*N*‐bis(4‐(9‐phenyl‐9*H*‐carbazol‐2‐yl)phenyl)aniline (**N‐OMe‐2‐Cb‐Ph**).

Yield: 47 %; ^1^H NMR (400 MHz, CDCl_3_) δ 8.16 (t, *J*=8.4 Hz, 29H), 7.66 – 7.58 (m, 59H), 7.52 (dd, *J*=18.8, 9.8 Hz, 74H), 7.41 (d, *J*=3.5 Hz, 30H), 7.34 – 7.27 (m, 16H), 7.23 (d, *J*=7.5 Hz, 5H), 7.11 (dd, *J*=17.9, 8.0 Hz, 50H), 7.01 – 6.95 (m, 3H), 6.87 (dd, *J*=8.9, 6.3 Hz, 17H), 3.82 (s, 25H); ^13^C NMR (400 MHz, CDCl_3_) δ 155.38, 146.95, 146.33, 146.12, 140.55, 140.40, 139.59, 139.40, 138.00, 136.65, 134.35, 128.91, 128.10, 127.06, 126.50, 126.25, 124.78, 122.20, 121.95, 121.16, 119.48, 119.19, 119.00, 118.20, 113.84, 108.71, 106.60, 54.47.

*N*‐(3′‐(9*H*‐carbazol‐9‐yl)‐[1,1′‐biphenyl]‐4‐yl)‐3′‐(9*H*‐carbazol‐9‐yl)‐*N*‐(4‐methoxyphenyl)‐[1,1′‐biphenyl]‐4‐amine (**N‐OMe‐p‐Cb**).

Yield: 88 %; ^1^H NMR (400 MHz, CDCl_3_) δ 8.17 (d, *J*=7.7 Hz, 4H), 7.78 (s, 2H), 7.70 – 7.61 (m, 4H), 7.51 (dd, *J*=18.1, 8.4 Hz, 10H), 7.42 (t, *J*=7.1 Hz, 4H), 7.30 (t, *J*=7.4 Hz, 4H), 7.17 (d, *J*=8.5 Hz, 6H), 6.90 (d, *J*=9.0 Hz, 2H), 3.82 (s, 3H); ^13^C NMR (101 MHz, CDCl_3_) δ 155.67, 146.62, 141.54, 139.86, 139.02, 137.17, 132.46, 129.18, 126.76, 124.92, 124.50, 124.11, 122.36, 121.96, 119.29, 118.90, 113.95, 108.81, 54.47.

### Soft‐Template Electropolymerization

The electrochemical experiments were performed using an Autolab potentiostat (Metrohm). Three electrodes were used: gold‐coated silicon wafers as working electrode, glassy carbon rod as counter‐electrode and saturated calomel electrode (SCE) as reference electrode. For investing the effect of water (H_2_O), two solvents were tested: dichloromethane (CH_2_Cl_2_) and dichloromethane saturated with water (CH_2_Cl_2_ + H_2_O sat.). The solubility of H_2_O in CH_2_Cl_2_ being very low,^[47]^ CH_2_Cl_2_ + H_2_O sat. was simply prepared by gently mixing CH_2_Cl_2_ and H_2_O and by keeping the organic phase. Bu_4_NClO_4_ and the monomer were added with a concentration of 0.1 M and 0.005 M, respectively. Using CH_2_Cl_2_ + H_2_O sat., it was demonstrated the formation of inverse micelles before electropolymerization.^[38]^ The presence of micelles was demonstrated even with close monomers.^[48]^

### Smooth Surfaces by Electropolymerization

Smooth surfaces were prepared at constant potential (CP) and using an ultra‐short deposition charges (1 mC cm^−2^) necessary to obtain very low roughness. However, the smooth surfaces were electrochemically reduced (1 back scan from E^ox^ to −1 V) in order to form the same polymers than by CV.

### Surface Characterization

The surface structures were imaged with a JEOL JSM‐6700F scanning electron microscope (SEM) after metallization. The surface wettability was characterized by measuring apparent contact angles (*θ*) using a KRÜSS DSA30 goniometer. Three probes of liquids of different surface tensions (γ_LV_) were used: water (72.8 mN/m), diiodomethane (50.0 mN/m) and *n*‐hexadecane (27.6 mN/m). Each value given here is a mean value of five measurements. For smooth surfaces, the apparent contact angles are called Young’ angles (*θ* ^Y^). Their surface energy *γ*_SV_ were calculated with the Owens‐Wendt equation as well as their polar (*γ*_SV,P_) and dispersive (*γ*_SV,D_) parts.

## Results and Discussion

### Electropolymerization and Electrochemical Characterization

**Figure 1:**
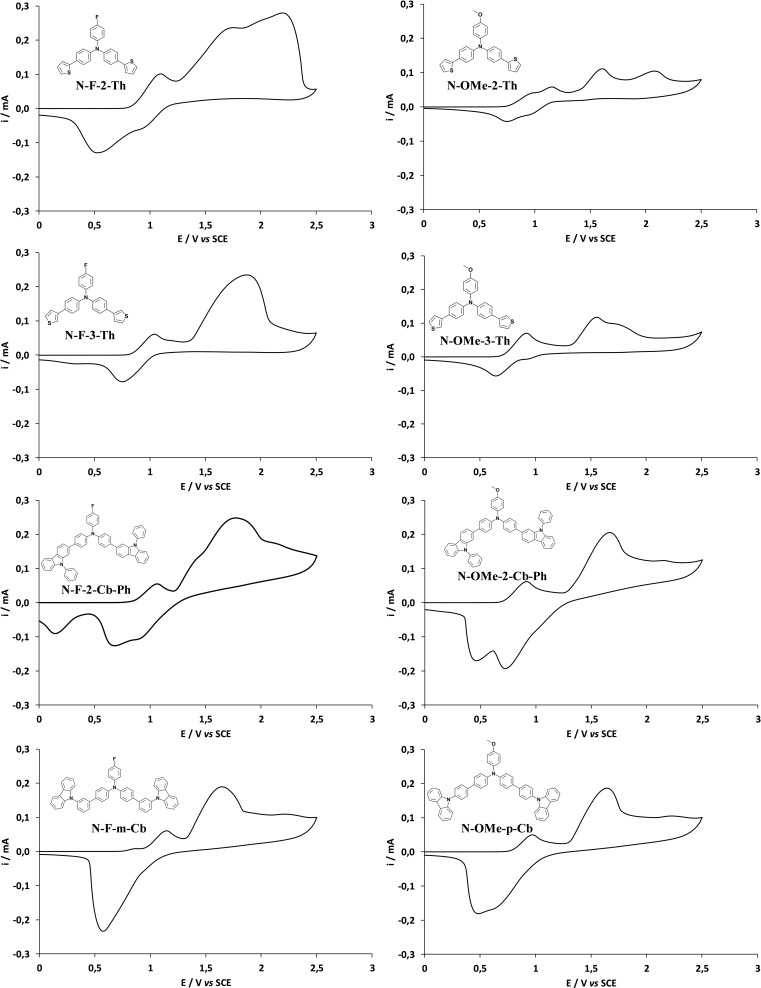
CV curves of each monomer (0.005 M) in CH_2_Cl_2_ with 0.1 M of Bu_4_NClO_4_. The potential range sweep was from 0 to 2.5 V, the scan rate was 100 mV s^–1^. The first scan is shown.

The cyclic voltammetry (CV) curves of each monomer are given in Figure 1. There are at least two important peaks. The peak of relatively low intensity at ≈ 1 V *vs* SCE observed in the CV of all monomers was attributed to triphenylamine oxidation.[^49^, ^50^] This peak was already present with tri‐substituted triphenylamine.^[41]^ For the carbazole derivatives, the oxidation peak of carbazole oxidation is also at ≈ 1 V and the preferential position is 3.[^51^, ^52^] The peak of higher intensity at ≈ 1.5‐1.8 V is the monomer oxidation potential (E^ox^) and was used for the depositions. These monomers being fully conjugated, their high intensity is due to the formation of energetically favorable radical cations. Peaks at higher potentials are also sometimes observed with these monomers. Indeed, even if these monomers are fully conjugated there are not perfectly planar and each monomer has a different planarity.^[41]^ π‐stacking interactions are maximal if the molecule is perfectly planar but, here, the plan is affected by the substituent especially by steric hindrance. For example, the steric hindrance is more important with carbazole in *ortho*‐position than in *para*‐position. For the thiophene derivatives, the oxidation peak of thiophene oxidation is also at ≈ 2 V and the preferential position is 2, and it is present in the CV curves.^[53]^ There are also more oxidation peaks in the CV of the thiophene derivatives than that of the carbazole ones.

For the back scans, the peaks are here relatively reversible. For the carbazole derivatives, their high intensity was assigned to the reduction of dicarbazole formed at 3 position.

**Figure 2:**
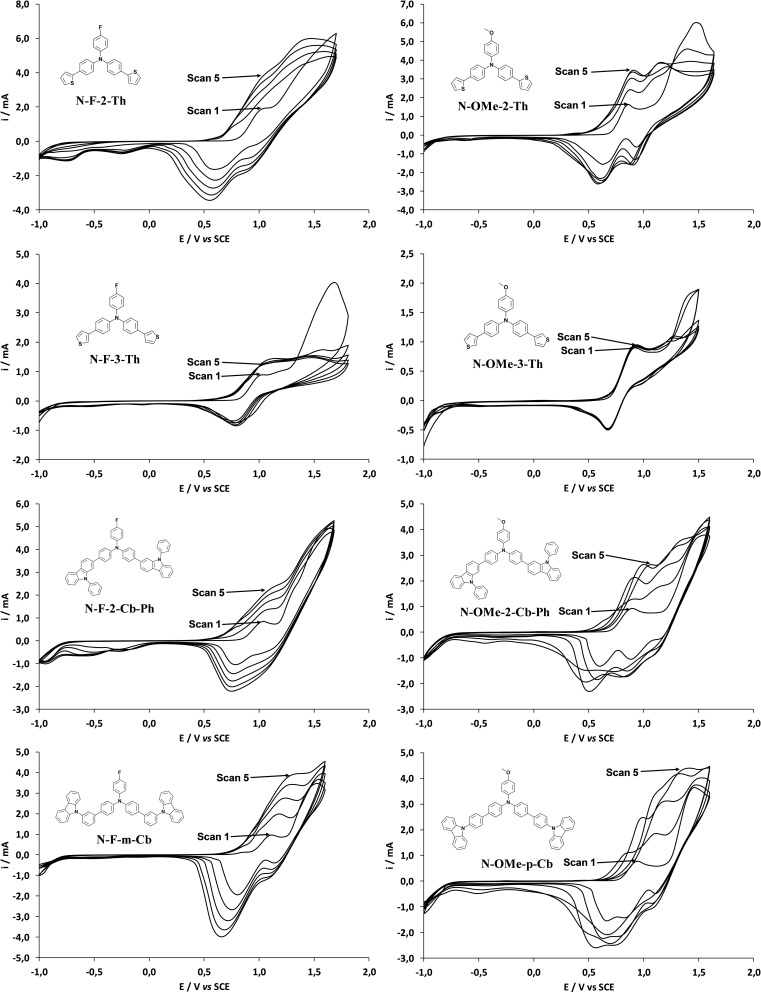
CV curves of each monomer (0.005 M) in CH_2_Cl_2_ with 0.1 M of Bu_4_NClO_4_. The potential range sweep was from −1 V to E^ox^, the scan rate is 20 mV s^–1^. The five scans are shown.

Then, depositions were first performed by CV in the two solvents. The potential range was from −1 V to E^ox^ in order to have both water oxidation and reduction, the scan rate was 20 mV s^−1^ and the number of deposition scans 1, 3 and 5. The CV curves in CH_2_Cl_2_ are shown in Figure 2. Excepted with the monomers with thiophene in 3‐position (**N‐F‐3‐Th** and **N‐OMe‐3‐Th**), it is observed an important increase of the oxidation and reduction after each scan indicating of excellent electrodeposition capacity due to the increase thickness deposition. With **N‐F‐3‐Th** and **N‐OMe‐3‐Th** the increase in intensity is much more reduced especially with **N‐OMe‐3‐Th**. For information, these monomers electropolymerized also perfectly in CH_2_Cl_2_ + H_2_O sat.

**Figure 3:**
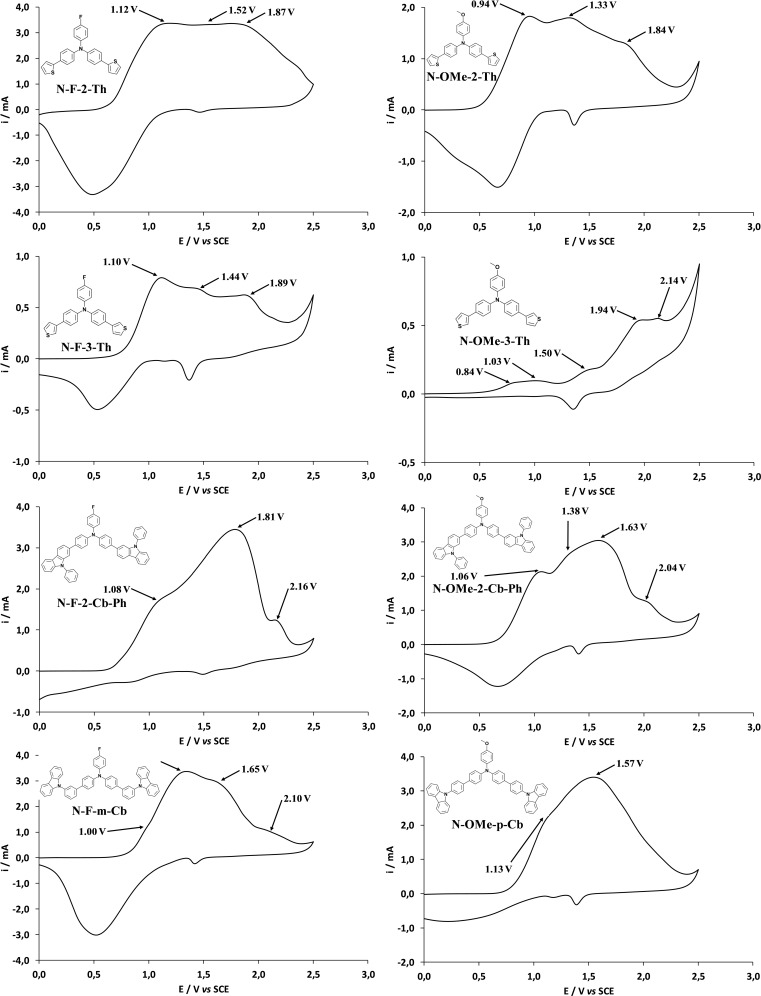
CV curves of each electrodeposited polymer in CH_2_Cl_2_ with 0.1 M of Bu_4_NClO_4_. The potential range sweep is from 0 to 2.5 V, scan rate is 20 mV s^–1^. The first scan is shown.

The electrodeposited polymer films were washed several times in CH_2_Cl_2_ and they were electrochemically analyzed in electrolyte free of monomers (Figure 3). These electrodeposited polymer films could be washed many times in CH_2_Cl_2_ without observing even a partial dissolution. This is probably due to strong π‐stacking interactions reducing their solubility. Indeed, these studied monomers have each a different solubility but they are the most soluble in CH_2_Cl_2_ or CHCl_3_.

First, the explanation of the results is not easy because in the CV of the monomers (Figure 1), a peak at ≈ 1 V is already present. Confirming previous works,[^41^, ^54^] the polymer CV curves show the peaks of the monomers (the peak at ≈ 1 V and the peak at E^ox^) indicating of its presence in the polymer films. However, with some monomers it is observed an important increase but especially the peak ≈ 1 V, which is probably due to the formation of oligomers. The proportion of oligomers *vs* monomers is different with each monomer. This is sometimes much higher with **F** group compared to **OMe** group (**N‐F‐3‐Th** and **N‐F‐m‐Cb**). This result is important because, if we want to favor the polymer growth in one‐direction, it is important to reduce the polymerization as much as possible. Indeed, the direction of the polymerization and the direction of π‐stacking interactions are not the same.

### Surface Properties of the Electrodepositions made by Cyclic Voltammetry (CV)

**Figure 4:**
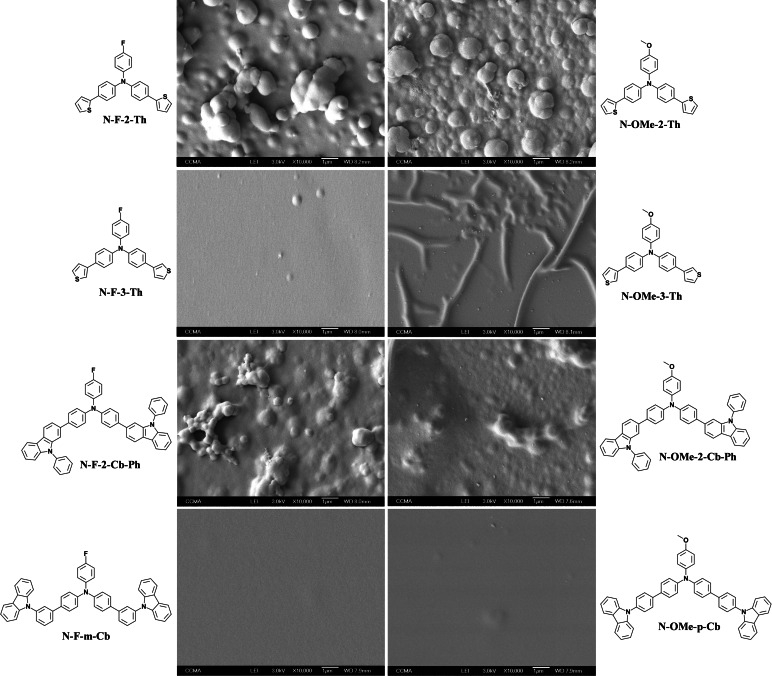
SEM images of each electrodeposited polymer in CH_2_Cl_2_ by CV (3 scans).

In the electrodeposited films in CH_2_Cl_2_ only non‐porous structures are observed. The films are either smooth or made of spherical particles indicating of usual three‐directional growth (Figure 4). The smoother films are obtained with **N‐F‐3‐Th**, **N‐OMe‐3‐Th**, **N‐F‐m‐Cb** and **N‐OMe‐p‐Cb**. The monomers **N‐F‐2‐Th**, **N‐OMe‐2‐Th**, **N‐F‐2‐Cb‐Ph** and **N‐OMe‐2‐Cb‐Ph** lead to spherical particles, which is due to a higher polymer insolubility in CH_2_Cl_2_.

**Figure 5:**
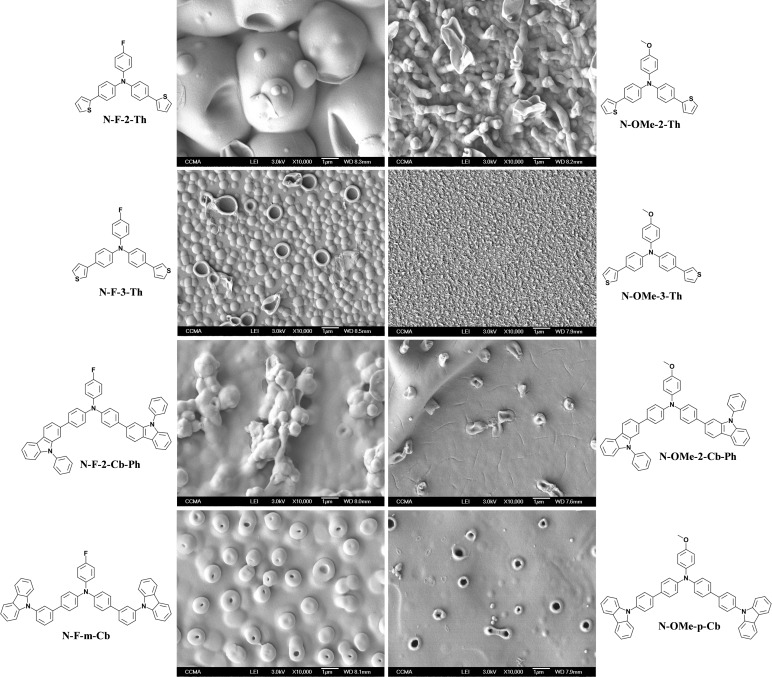
SEM images of each electrodeposited polymer in CH_2_Cl_2_ + H_2_O sat. by CV (3 scans).

**Figure 6:**
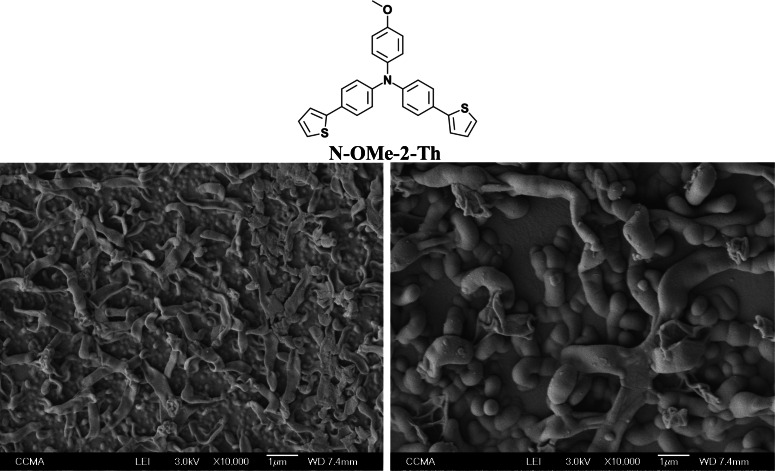
SEM images of **N‐OMe‐2‐Th** in CH_2_Cl_2_ + H_2_O sat. by CV (1 scan and 5 scans).

In CH_2_Cl_2_ + H_2_O sat., with **N‐F‐2‐Th**, very large gas bubbles are present on the substrate and the structures formed are close (Figure 5). Nanotubes with large diameters are observed **N‐F‐2‐Th** and **N‐OMe‐p‐Cb**. With **N‐F‐m‐Cb** densely packed nanotubes are obtained even if the structures are not very open. With **N‐OMe‐2‐Th**, the nanotubes are longer. We have added Figure 6 to better see the increase also in nanotube size with the number of scans.

The surface the most hydrophobic obtained by CV is the surface obtained in CH_2_Cl_2_ and with **N‐F‐2‐Th** (*θ*_w_=132.9° after 5 scans) that means a surface with spherical nanoparticles (Table 1). In CH_2_Cl_2_ + H_2_O sat., the surface the most hydrophobic was obtained with **N‐F‐2‐Cb‐Ph** (*θ*_w_=117.5° after 5 scans). All these surfaces are also superoleophilic with hexadecane.

**Table 1:** Apparent contact angles of each electrodeposited polymer prepared by CV.

Monomer	Number of scans	Solvent: CH_2_Cl_2_			Solvent: CH_2_Cl_2_ + H_2_O sat.		
		*θ*_w_ (°)	*θ*_Diiod_ (°)	*θ*_Hexa_ (°)	*θ*_w_ (°)	*θ*_Diiod_ (°)	*θ*_Hexa_ (°)
**N‐F‐2‐Th**	1 scan	87.0±7.2	28.6±1.3	<10	78.9±2.8	28.2±1.0	<10
	3 scans	122.2±5.1	10.9±2.2	<10	80.1±1.8	22.3±2.5	<10
	5 scans	**132.9**±2.5	22.2±1.2	<10	88.1±3.6	40.1±3.3	<10
**N‐F‐3‐Th**	1 scan	87.7±0.7	17.7±3.9	<10	81.1±2.7	16.7±2.6	<10
	3 scans	92.1±1.2	21.8±2.3	<10	100.9±1.1	15.1±2.4	<10
	5 scans	95.2±1.0	22.5±3.1	<10	96.5±2.0	<10	<10
**N‐F‐2‐Cb‐Ph**	1 scan	81.3±2.2	12.4±1.0	<10	97.3±2.5	27.1±5.4	<10
	3 scans	107.5±2.2	15.6±1.6	<10	98.1±0.8	<10	<10
	5 scans	110.7±9.3	14.0±1.8	<10	117.5±9.5	11.5±4.4	<10
**N‐F‐m‐Cb**	1 scan	89.1±2.1	21.2±1.3	<10	94.5±2.9	<10	<10
	3 scans	88.6±1.6	20.6±0.5	<10	108.2±1.2	<10	<10
	5 scans	103.7±1.4	34.3±6.5	<10	107.3±2.6	<10	<10
**N‐OMe‐2‐Th**	1 scan	72.5±3.8	35.9±0.5	<10	63.1±5.2	25.9±2.4	<10
	3 scans	95.5±2.0	46.7±4.6	<10	110.6±1.3	<10	<10
	5 scans	71.5±1.0	52.0±1.6	<10	80.2±2.4	29.0±4.3	<10
**N‐OMe‐3‐Th**	1 scan	63.6±1.4	40.0±0.7	<10	51.6±3.7	36.0±1.9	<10
	3 scans	75.5±2.0	31.1±1.2	<10	64.4±2.4	22.1±4.7	<10
	5 scans	57.1±4.2	45.8±1.7	<10	55.8±1.4	27.2±4.7	<10
**N‐OMe‐2‐Cb‐Ph**	1 scans	66.2±2.5	<10	<10	65.3±4.1	23.6±1.8	<10
	3 scans	85.4±2.5	26.7±1.3	<10	76.2±2.0	14.0±4.4	<10
	5 scans	60.5±2.3	25.8±3.3	<10	73.1±1.9	<10	<10
**N‐OMe‐p‐Cb**	1 scan	75.3±1.3	29.9±1.4	<10	72.5±1.7	28.7±2.0	<10
	3 scans	85.6±7.2	19.2±5.3	<10	82.2±2.7	26.4±3.2	<10
	5 scans	75.0±4.8	23.6±1.1	<10	74.0±1.2	26.7±4.8	<10

In order to explain these results, smooth surfaces with each monomer were also prepared because the equations explaining the effect of the surface roughness (Wenzel and Cassie‐Baxter equations)[^55^, ^56^] on the apparent contact angles depend on these angles also called Young's angles (*θ* ^Y^).^[57]^ On these smooth surfaces, the surface energy *γ*_SV_ as well as their disperse (*γ*_SV,D_) and polar parts (*γ*_SV,P_) were also determined using the Owens‐Wendt equation.^[58]^ All these polymer surfaces are intrinsically hydrophilic with 60.0° < *θ* ^Y^_w_ <78.9° and with 36.9 < *γ*_SV_ <46.5 mN m^−1^ (Table 2). As expected, the polymers with OMe groups are more hydrophilic than that with F groups. Hence, the results with high hydrophobicity can be accepted only with the Cassie‐Baxter equations indicating the presence of air between the water droplet and the rough surfaces. By contrast, the surfaces are superoleophilic with hexadecane because this liquid fully wetted the rough surfaces (Wenzel equation).

**Table 2:** Apparent contact angles and surface energy of electrodeposited smooth polymer.

Monomer	*θ* ^Y^_w_ (°)	*θ* ^Y^_Diiod_ (°)	*θ* ^Y^_Hexa_ (°)	*γ*_SV_	*γ*_SV,D_	*γ*_SV,P_
**N‐F‐2‐Th**	70.4±3.3	33.7±3.2	<10	41.1	30.8	10.3
**N‐F‐3‐Th**	65.0±2.5	33.8±2.5	<10	43.8	30.2	13.6
**N‐F‐2‐Cb‐Ph**	69.3±3.1	31.5±6.5	<10	41.9	31.1	10.8
**N‐F‐m‐Cb**	78.9±4.0	38.7±7.1	<10	36.9	30.7	6.2
**N‐OMe‐2‐Th**	60.0±1.6	34.2±3.8	<10	46.5	29.6	16.9
**N‐OMe‐3‐Th**	60.9±5.2	31.0±4.1	<10	46.4	30.3	16.1
**N‐OMe‐2‐Cb‐Ph**	63.7±3.1	20.1±5.1	<10	44.8	30.6	14.2
**N‐OMe‐p‐Cb**	65.9±1.2	29.5±2.0	<10	43.8	31.1	12.7

### Surface Properties of the Electrodepositions made at Constant Potential (CP)

**Figure 7:**
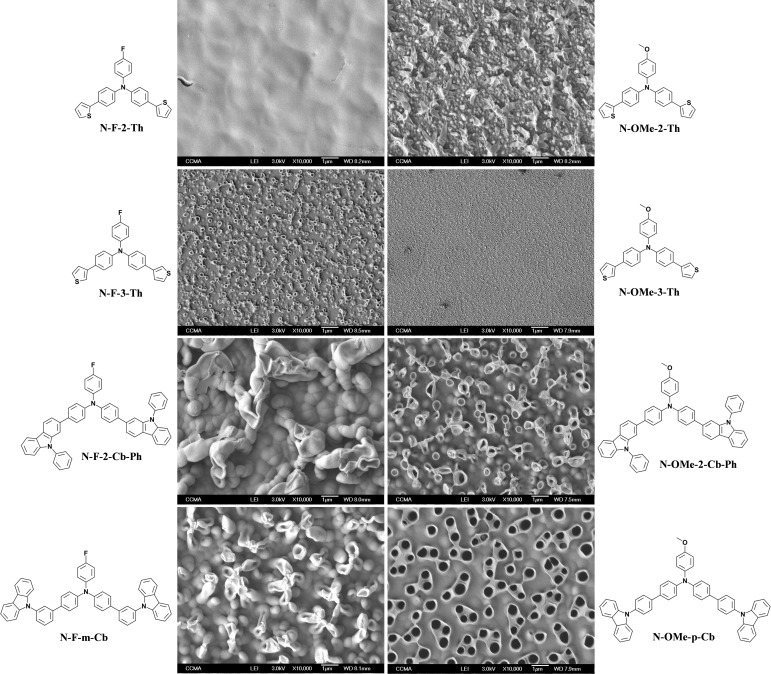
SEM images of each electrodeposited polymer in CH_2_Cl_2_ + H_2_O sat. at CP (100 mC cm^−2^).

At CP, the structures formed are much more homogeneous (Figure 7). This is expected because using a potentiostatic method, only water oxidation is possible (2H_2_O→O_2_ + 4H^+^ + 4e^–^) and the released of gas bubbles is more controlled. By CV, both water oxidation and water reduction are present but not at the same potential ≈ 2 V and −0.5 V respectively.

**Figure 8:**
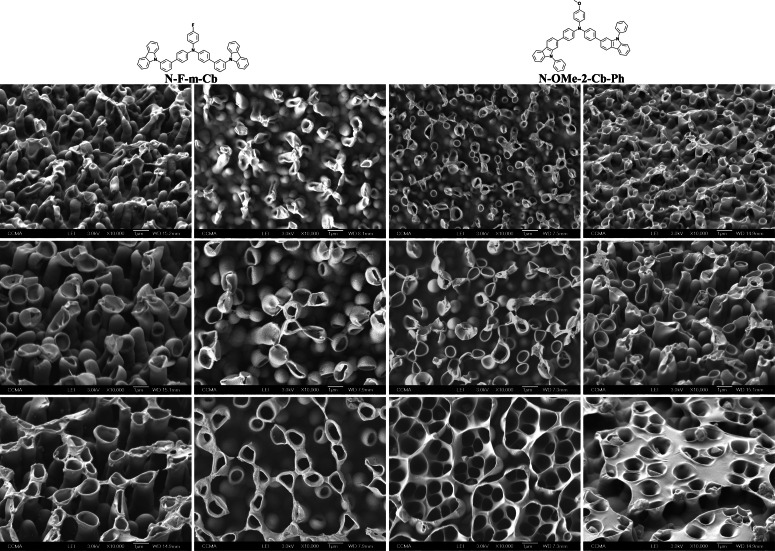
SEM images of **N‐F‐m‐Cb** and **N‐OMe‐2‐Cb‐Ph** in CH_2_Cl_2_ + H_2_O sat. at CP (100, 200 and 400 mC cm^−2^). Pictures obtained without or with a surface inclination of 45°.

Nanotubes are obtained excepted with **N‐F‐2‐Th** probably due to a higher solubility with this monomer. With the thiophene derivatives, densely‐packed nanotubes of extremely small diameter are formed while with **N‐OMe‐2‐Th** longer tubes. With the carbazole derivatives the tubes are much longer. It is observed an increase of the size of the tubes and also their diameter, as the deposition charge increases (Figure 8). When the diameter of the tubes become critical, it is observed the merge of the tubes leading to nanomembranes, which are particularly rare in literature with this process.^[59]^

The surfaces are less hydrophobic (Table 3), which is expected because at CP the polymers formed are doped with, here, relatively hydrophilic perchlorate ions (ClO_4_^−^). The highest hydrophobicity was obtained with **N‐F‐3‐Th** at high deposition charge (*θ*_w_=106.5° for 200 mC cm^−2^), that means with nanotubes of extremely small size. With monomers leading to nanomembranes, it is observed a decrease of hydrophobicity as the deposition charge increases. A decrease is observed after because the diameter of the tubes becomes too large leading to a wetting inside the tubes (Wenzel equation).

**Table 3:** Apparent contact angles of each electrodeposited polymer prepared at CP.

Monomer	Deposition charge (mC cm^−2^)	Solvent: CH_2_Cl_2_			Solvent: CH_2_Cl_2_ + H_2_O sat.		
		*θ*_w_ (°)	*θ*_Diiod_ (°)	*θ*_Hexa_ (°)	*θ*_w_ (°)	*θ*_Diiod_ (°)	*θ*_Hexa_ (°)
**N‐F‐2‐Th**	12.5	70.1±2.7	27.6±1.1	<10	78.6±2.6	34.5±6.7	<10
	25	73.2±0.7	26.6±0.5	<10	83.5±5.3	30.0±8.0	<10
	50	71.4±2.5	40.3±1.0	<10	64.5±2.5	38.2±2.3	<10
	100	76.6±9.0	<10	<10	52.7±4.9	28.5±0.8	<10
	200	88.1±8.5	<10	<10	55.0±4.7	37.3±6.0	<10
	400	86.7±1.8	<10	<10	60.0±3.8	43.6±0.5	<10
**N‐F‐3‐Th**	12.5	85.7±1.2	19.2±0.2	<10	87.3±2.1	21.8±7.5	<10
	25	83.0±3.8	23.9±1.0	<10	78.4±0.6	21.2±7.0	<10
	50	76.9±4.1	23.8±5.0	<10	87.1±1.5	23.7±7.6	<10
	100	87.4±0.5	13.3±0.5	<10	99.8±0.4	24.0±0.8	<10
	200	83.6±2.8	20.2±1.7	<10	**105.3**±0.8	19.4±1.0	<10
	400	83.5±2.1	16.6±1.5	<10	**106.5**±3.5	38.1±4.6	<10
**N‐F‐2‐Cb‐Ph**	12.5	68.2±2.3	26.1±5.3	<10	55.4±1.3	29.9±4.9	<10
	25	65.8±6.4	32.6±2.2	<10	64.1±1.5	15.5±2.7	<10
	50	54.5±1.0	22.7±2.4	<10	65.8±2.0	10.3±2.2	<10
	100	61.5±2.0	21.8±5.1	<10	63.3±2.9	16.6±2.9	<10
	200	48.9±1.2	19.0±4.4	<10	71.3±5.4	19.8±6.5	<10
	400	46.4±1.1	21.9±5.0	<10	77.3±3.4	28.9±7.0	<10
**N‐F‐m‐Cb**	12.5	82.6±0.5	19.0±1.1	<10	70.2±0.4	14.6±4.1	<10
	25	74.9±3.0	28.0±2.4	<10	60.9±1.7	14.8±4.1	<10
	50	69.5±2.6	24.9±3.5	<10	101.7±1.8	13.1±1.4	<10
	100	73.0±3.2	18.7±3.9	<10	77.8±3.3	12.2±2.4	<10
	200	75.8±3.0	27.9±2.8	<10	87.9±4.7	17.0±3.2	<10
	400	64.8±1.7	25.4±1.6	<10	85.1±5.63	34.8±5.3	<10
**N‐OMe‐2‐Th**	12.5	49.6±1.7	34.2±1.0	<10	46.1±3.4	37.9±2.8	<10
	25	32.4±3.2	35.4±0.5	<10	46.6±0.7	45.3±2.0	<10
	50	45.0±1.7	41.4±2.7	<10	50.6±0.9	55.2±3.1	27.8±3.2
	100	61.3±0.8	30.0±1.7	<10	48.0±2.6	**93.2**±0.7	**76.2**±0.8
	200	63.8±3.0	33.3±0.4	<10	46.2±1.2	63.6±5.5	19.2±2.5
	400	66.8±1.7	78.5±4.6	48.3±2.5	58.2±3.7	60.7±0.4	24.8±2.0
**N‐OMe‐3‐Th**	12.5	54.7±3.8	34.4±1.4	<10	43.6±3.9	36.7±1.1	<10
	25	52.7±0.9	38.0±6.0	18.8±1.0	48.7±7.4	35.8±3.1	<10
	50	57.1±0.6	34.7±6.3	<10	64.9±1.8	36.4±3.1	<10
	100	71.1±0.9	19.6±2.0	<10	63.1±1.8	34.4±1.1	<10
	200	60.5±0.9	27.9±0.6	<10	66.0±3.3	37.1±2.6	<10
	400	62.4±0.5	28.2±1.0	<10	51.2±1.9	29.7±4.0	<10
**N‐OMe‐2‐Cb‐Ph**	12.5	48.7±4.6	19.0±2.3	<10	57.1±0.6	<10	<10
	25	51.5±2.2	22.9±5.2	<10	73.5±3.3	<10	<10
	50	57.2±2.2	17.6±2.5	<10	66.0±3.6	28.0±0.7	<10
	100	51.0±1.5	<10	<10	53.7±1.4	<10	<10
	200	51.7±1.1	15.1±4.6	<10	38.4±1.6	<10	<10
	400	51.8±4.4	<10	<10	34.8±3.0	<10	<10
**N‐OMe‐p‐Cb**	12.5	68.0±0.3	25.9±1.9	<10	59.8±1.0	30.0±1.2	<10
	25	67.2±0.8	30.3±2.5	<10	65.3±3.0	18.3±1.9	<10
	50	56.1±2.4	30.7±1.0	<10	65.4±1.1	28.9±0.6	<10
	100	63.6±1.4	23.7±1.2	<10	70.6±0.8	23.4±1.2	<10
	200	61.3±3.4	25.7±3.9	<10	74.0±4.9	33.7±0.7	<10
	400	55.5±1.4	35.1±6.5	<10	38.6±3.7	33.1±1.2	<10

With **N‐OMe‐2‐Th**, the surfaces are both hydrophilic and oleophobic (*θ*_w_=48.0° and *θ*_hexa_=76.2° for 100 mC cm^−2^), indicating a higher air fraction, between the rough surface and the liquid droplet, with hexadecane than with water. This is extremely surprising because water has a much higher surface tension than hexadecane (*γ*_L,w_=72.8 and 27.6 mN m^−1^, respectively). These wettability results indicated than the polymer is both extremely hydrophilic and oleophobic.

## Conclusions

Here, we investigated a soft‐template electropolymerization approach in micellar condition for forming porous nanostructures such as nanotubes. Monomers highly favoring π‐stacking interactions are particularly interesting. Original monomers highly conjugated were synthesized from a triphenylamine building block. All these monomers had high electrodeposition capacity except the monomers with thiophene in 3‐position. In the electrodeposited films, electrochemical analyses confirmed the presence of monomers but with different proportions as a function of the monomer. More ordered structures were obtained at constant potential. With some of these investigated monomers, densely packed nanotubes were formed and their merger at high deposition charge, leading to nanomembranes. Their surface hydrophobicity and oleophobicity were also extremely various. These nanomembranes could be used in the future in oil/water separation membranes.

## Author Contributions

Khady Diouf, Formal analysis. Alioune Diouf: Supervision, Resources, Funding acquisition. Abdoulaye Dramé: Supervision, Resources, Funding acquisition. Frédéric Guittard: Supervision, Resources, Funding acquisition. Thierry Darmanin: Validation, Writing – original draft.

## Conflict of Interests

The authors declare no conflict of interest.

## Data Availability

The data that support the findings of this study are available from the corresponding author upon reasonable request.
